# Editorial: The Potential of Fungi for Enhancing Crops and Forestry Systems

**DOI:** 10.3389/fmicb.2021.813051

**Published:** 2021-12-22

**Authors:** Samantha C. Karunarathna, Nanjappa Ashwath, Rajesh Jeewon

**Affiliations:** ^1^Center for Mountain Futures, Kunming Institute of Botany, Chinese Academy of Science, Kunming, China; ^2^Institute for Future Farming Systems, School of Medical and Applied Sciences, Central Queensland University, Rockhampton, QLD, Australia; ^3^Department of Health Sciences, Faculty of Medicine and Health Sciences, University of Mauritius, Moka, Mauritius

**Keywords:** agriculture, biofertilizers, biopesticides, biotechnology, endophytes, *Fusarium*, mycorrhizal fungi, phytohormones

In agricultural lands, long-term chemical inputs for the purpose of disease control result in both chronic and acute negative effects, such as environment pollution, human health complications, and spray-resistant pest populations (Aktar et al., 2009). For sustainable agriculture, biotechnological products are developed using targeted fungal species (Bamisile et al., 2021). Members in the genera *Alternaria, Aspergillus, Chaetomium, Fusarium, Penicillium, Serendipita, Phoma*, and *Trichoderma* are known as plant growth-promoting fungi, and they have potential to be developed further as biofertilizers (Hyde et al., 2019). In addition, some biofertilizers act as antagonists and suppress incidents of soil-borne plant pathogens while helping in the biocontrol of plant diseases (Pirttilä et al., 2021). Past research has shown that endophytic fungi can be successfully used as plant defenders, growth promoters, and competitors of microbial pathogens, which has potential for utilization in a wide variety of medical, agricultural and industrial fields. This is largely owing to their ubiquitous distribution as symbionts associated with many different plants (Stone et al., 2000; Scannerini et al., 2001; Strobel, 2003; Rania et al., 2016).

Ectomycorrhizae improve soil structure and nutrients, protect plants against root pathogens, promote plant growth by producing phytohormones, improve the survival and growth of seedlings, and increase the photosynthetic rate of plants (Santoyo et al., 2021). Ectomycorrhizae also cut down fertilization costs in an environmentally friendly manner. These fungi are important for the growth enhancement of economically important crops including trees belonging to genera *Castanopsis, Dipterocarpus, Eucalyptus, Fagus, Picea, Pinus, Quercus*, and *Shorea*. *Cenocococum, Laccaria, Pisolithus, Rhizopogon, Russula, Scleroderma*, and *Thelephora* are well-known ectomycorrzhiza genera that increase the rate of survival and growth of eucalyptus, pine and oak seedlings in both plantations and reforestation programs (Hyde et al., 2019).

Today, the floriculture trade faces extinction threats because of habitat loss and over-exploitation of attractive species (Benton et al., 2021). Mycorrhizal and endophyte fungi are shown to stimulate seed germination and disease control (Domka et al., 2019). Studies have shown the role of endophytic fungi in enhancing plant vigor in both normal and stressful environments (Fadiji and Babalola, 2020). Several fungal species produce a variety of bioactive compounds that play important roles in the physiological activities of the host plant, influencing the growth of hosts (Sarkar et al., 2021). Members of fungal genera like *Aspergillus, Botrytis, Cercospora, Penicillium*, and *Rhizopus* produce important plant growth hormones (Shi et al., 2017). As a response to the importance of fungi in enhancing plant growth, we proposed the Research Topic “*The Potential of Fungi for Enhancing Crops and Forestry Systems*.” In this Research Topic, we have collected six original research articles that would enable mycologists to gain better insights into the use of fungi in agriculture. We are very thankful to all authors who have contributed to this Research Topic.

Global production of muskmelon (*Cucumis melo*) is affected by gummy stem blight and wilt that causes enormous losses. Nuangmek et al. discuss the isolation and characterization of a new endophytic fungus, *Trichoderma phayaoense* from the leaves of the Siam weed. Findings of this research indicate that *T. phayaoense* can control muskmelon pathogens, *F. equiseti* and *S. cucurbitacearum in vitro*. Furthermore, *T. phayaoense* shows the antagonistic effects against gummy stem blight and *Fusarium* wilt in muskmelon seedlings while *T. phayaoense* also shows improvements in plant and fruit development. *Trichoderma* biopriming improves rice cultivation in drought-stressed soils by activating various plant metabolic pathways. Bashyal et al., conclude that *T. harzianum* biopriming delays drought stress in rice by a multitude of molecular programming. Truffle fungi are well known for the most widely cultivated edible ectomycorrhizal fungi. Grupe et al. present a successful method for cultivating *Tuber lyonii*, the pecan truffle, on pecan (*Carya illinoinensis*) seedlings in a field setting. The optimal way of truffle propagation is suggested as transplanting inoculated pecan seedlings after 2 or 3 years post-inoculation. *Rhizopus oryzae* is an important and devastating post-harvest pathogen causing tobacco leaf mildew disease during flue-cured tobacco period. After successful screening of 36 bacteria and one fungus, Pan et al. demonstrate that candidate bacterial strain *B. amyloliquefaciens* B9601-Y2 is a potential antagonist for the management of tobacco leaf mildew during flue-curing. Pine wilt disease (PWD) is well-known as a fatal disease to pines (*Pinus* spp.) worldwide. Chu et al. shows that exogenous root ectomycorrhizal fungi/ dark septate endophytic fungi inoculation increases *Pinus tabulaeformis* resistance to PWD by improving the rhizosphere microenvironment. Soil fungi play a vital role in solubilizing of insoluble minerals in the soil and supply them to plants. Khuna et al. describe and discuss the mineral-solubilizing ability of three new fungi species viz. *A. chiangmaiensis, A. pseudopiperis*, and *A. pseudotubingensis* in northern Thailand. Further, the study shows that the three fungi enhance the plant growth in both *Arabidopsis* and onion plants.

In summary, this Research Topic published six scientific contributions which consolidate and expand our knowledge on the recent advances in fungi for enhancing crops and forestry systems and how they are useful for eco-friendly crops and forestry systems. We sincerely hope that more scientists will be inspired and encouraged by this Research Topic to make their contributions to improve plant growth using fungi especially in economically important crops.

## Author Contributions

SK drafted the editorial. All authors contributed to editorial revision and approved the final paper.

## Funding

SK thank CAS President's International Fellowship Initiative (PIFI) young staff under the (grant number: 2020FYC0002), National Science Foundation of China (NSFC) project (code31851110759), Kunming Institute of Botany, and Chinese Academy of Science.

## Conflict of Interest

The authors declare that the research was conducted in the absence of any commercial or financial relationships that could be construed as a potential conflict of interest.

## Publisher's Note

All claims expressed in this article are solely those of the authors and do not necessarily represent those of their affiliated organizations, or those of the publisher, the editors and the reviewers. Any product that may be evaluated in this article, or claim that may be made by its manufacturer, is not guaranteed or endorsed by the publisher.
